# Role of Mitochondrial Dynamics in Skin Homeostasis: An Update

**DOI:** 10.3390/ijms26051803

**Published:** 2025-02-20

**Authors:** Tao Quan, Ran Li, Ting Gao

**Affiliations:** College of Veterinary Medicine, China Agricultural University, Beijing 100083, China; s20243051072@cau.edu.cn (T.Q.); 18246269672@163.com (R.L.)

**Keywords:** ultraviolet radiation, skin aging, mitochondria, oxidative stress

## Abstract

Skin aging is the most prominent phenotype of host aging and is the consequence of a combination of genes and environment. Improving skin aging is essential for maintaining the healthy physiological function of the skin and the mental health of the human body. Mitochondria are vital organelles that play important roles in cellular mechanisms, including energy production and free radical balance. However, mitochondrial metabolism, mitochondrial dynamics, biogenesis, and degradation processes vary greatly in various cells in the skin. It is well known that mitochondrial dysfunction can promote the aging and its associated diseases of the skin, resulting in the damage of skin physiology and the occurrence of skin pathology. In this review, we summarize the important role of mitochondria in various skin cells, review the cellular responses to vital steps in mitochondrial quality regulation, mitochondrial dynamics, mitochondrial biogenesis, and mitochondrial phagocytosis, and describe their importance and specific pathways in skin aging.

## 1. Introduction

Cellular aging is a complex physiological process characterized by an eternal inhibition of proliferation [1]. Numerous data show that cellular senescence is accompanied by a gradual decrease in the ability to proliferate and differentiate, eventually becoming physiologically nonfunctional [2]. It is well known that skin aging is the most obvious feature of organismal aging. In addition to physiological aging caused by endogenous factors such as aging, exogenous factors including ultraviolet radiation further aggravate skin aging [3]. Clinically, the phenotypes of skin aging include skin thinning, wrinkle appearance, hyperpigmentation, and capillary dilation with enlarged pores [4]. Skin aging not only changes the host’s appearance and brings serious psychological burden, which in turn hinders normal work and social interactions, but also leads to the occurrence of related diseases and even skin cancer [5,6]. Current research has confirmed the importance of mitochondrial functional homeostasis in skin health and the key role that mitochondrial dysfunction plays in skin inflammation, skin diseases, and skin cancers. Mitochondrial dysfunction in the skin and other tissues is mechanistically based on excessive ROS production and its induction of oxidative stress, although some specific skin injuries are mediated by other mitochondrial pathways [7]. Although scientists have made many breakthroughs in revealing mitochondria-associated genes and proteins that play important roles in health- and disease-related environments, it is still difficult to unify views of how mitochondrial dysfunction drives the changes associated with skin aging [8].

Here, we not only discuss the critical role mitochondria play in healthy skin physiology versus premature skin aging and the associated diseases that result, but we also discuss the newly proposed idea that mitochondrial dysfunction promotes the pathogenesis of age-related skin diseases. Specifically, therapeutic strategies to ameliorate mitochondrial dysfunction and thereby slow skin aging by targeting mitochondrial dynamics, mitochondrial biogenesis, and mitochondrial autophagy are argued to be beneficial to humans in terms of healthy aging, prevention of age-associated diseases, and longevity.

## 2. Skin Structure

The skin is the outer sheath of the body, which is in contact with the external environment. It is a complex tissue that consists of several layers of tissue, each of which is made up of different cells. It has important functions such as sensory, thermal insulation, and protection against water loss, and is a physical barrier against pathogens [7].

The skin is made up of more than 20 different types of cells and is divided into three layers: epidermis, dermis, and subcutaneous. The epidermis forms the outermost layer, offering a defensive barrier to the skin [9]. The epidermis is mainly differentiated from keratinocytes. Originating from the ectoderm during embryogenesis, keratinocytes form the main cell type in the epidermis and form the protective layer of the skin. Upon differentiation as they move from the basal layer to the surface of the skin, keratinocytes undergo morphological changes and produce keratin, which contributes to skin strength and waterproofing [10,11].

The dermis is located under the epidermis and is connected to the epidermis by the dermal-epidermal connection and contains a variety of structures, including blood vessels, sebaceous glands, sweat glands, etc. Dermal-epidermal junction (DEJ), which consists of an abundance of extracellular matrix (ECM) and various functional structures. In the compartments of the skin, the dermis has the richest cellular richness. Among them, fibroblasts are the main cell type in the dermis and exert a central role in the synthesis and secretion of the ECM, which is composed of structurally related proteins including collagen and elastin, glycoproteins, and glycosaminoglycans. Overall, these components maintain the elasticity, fullness, and resilience of the skin [10,12,13,14]. Other cells are also present in the dermis, including mesenchymal stem cells (MSCs), fat cells, and immune cells, the latter of which are less numerous due to the presence of blood vessels [15]. In addition, the dermal chamber is home to additional functionally complex ingredients, including hair follicles, sweat glands, sebaceous glands, and sensory nerve endings [14].

Finally, the hypodermis is the innermost layer of skin [14]. It is mainly constituted of adipocytes, preadipocytes, and stem cells derived from adipose but is also composed of fibroblasts, macrophages, T cells, and erythrocytes, forming a network of interstitial vascular cells in nerves, muscles, and hair follicles [16]. The central role of the subcutaneous tissue is to link the skin to substructures, including muscles and bones, offering a pillar and insulation to the host and being regarded as a permanent energy storage chamber [17].

## 3. Skin Aging

**Figure 1:**
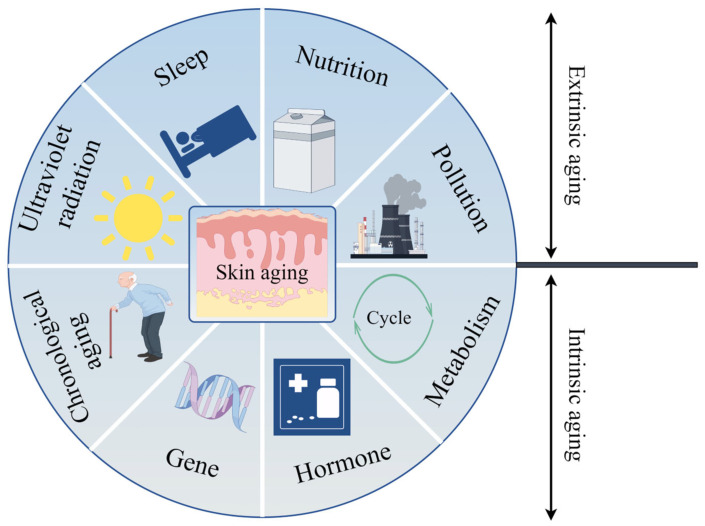
Skin aging. Skin aging is divided into endogenous and exogenous aging. Intrinsic skin aging is mainly determined by chronological aging, genes, hormones, and metabolism and is the natural result of changes in the physiological system over time, while external aging is caused by a combination of external environments, including ultraviolet radiation, sleep, nutrition, and pollution.

Acts as a barrier between the body and the external environment, the vital location of the skin determines the important function of keeping balance in the body and maintaining the survival of the host [18]. This leads to the skin, the organ most susceptible to environmental elements, including pollutants, temperature, and sun exposure. Physical and chemical responses induced by a variety of internal and external factors can lead to a gradual deficiency of skin structure and function, ultimately resulting in skin aging [19]. Intrinsic aging is mainly determined by genes and is the natural result of alterations in the physiological system over time; however, external aging is caused by a combination of external environments [20], including ultraviolet (UV) exposure, smoke, pollutants, nutrients, and exercise (Figure 1). Clinical phenotypes are associated with skin aging, including dry and rough, wrinkle formation, decreased elasticity, no regular pigment distribution, and a pronounced vascular system [21].

Ultraviolet exposure is the most common external factor that induces skin aging [22,23]. Ultraviolet radiation (UVR) exposure is an important environmental element that induces apoptosis and skin barrier damage [24]. Of the three subcategories of UVR, UVA (320–400 nm) and UVB (280–320 nm) reach earth in large numbers, while UVC (200–280 nm) does not [25]. As a result, UVA and UVB bear responsibility for most UVR-related health damages [25]. UVA is a more extensive ingredient that penetrates the dermis, while UVB, which typically does not exceed 10% of UVR, has high energy and is mainly assimilated by epidermal cells [25,26].

Senescence is often defined as the slow decline of an organism’s functions over time, which can be induced by the synchronous deterioration of interrelated cellular capacities. Although often mistaken for being found only in modern humans and animals in function, in fact, most metazoans indicate various markers of aging during their lifetime [27]. There is a great deal of interest in understanding aging and aging-related conditions and their associated molecular mechanisms in the hope of delaying these alterations. Researchers have long struggled to explore the process of aging. Especially if aging becomes apparent long after the reduced fertility, and therefore the power of nature has long since been weakened, how does the genetic process of aging work?

## 4. Mitochondria and Skin

**Figure 2:**
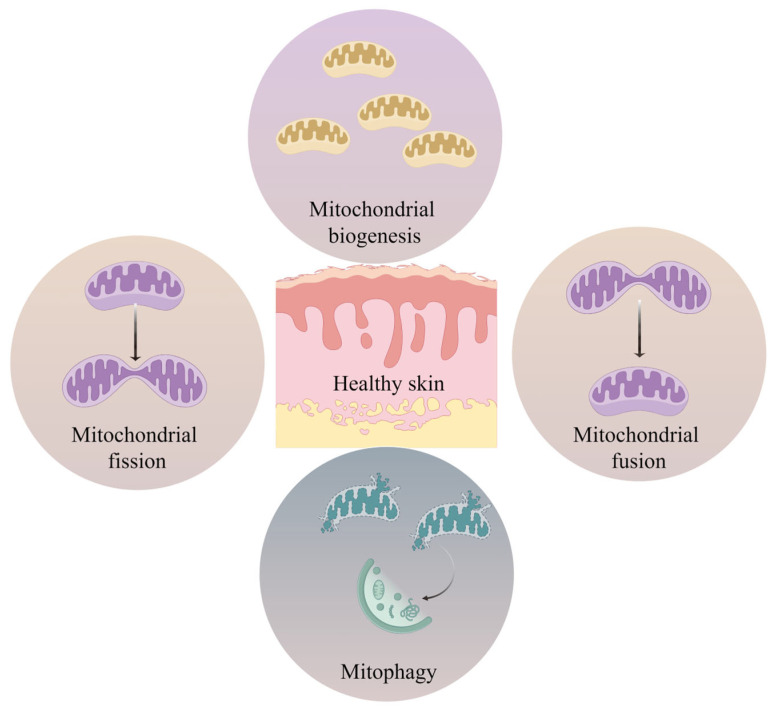
Skin health is tied to mitochondrial homeostasis. The state of healthy skin is associated with dynamic mitochondrial quality control, including mitochondrial biogenesis, mitochondrial fusion, mitochondrial division, and mitochondrial autophagy.

Mitochondria are found in almost all eukaryotic cells, and they are multifunctional organelles. Mitochondria exert a vital role in signaling, including bioenergetics, reactive oxygen species (ROS) production, catabolism and anabolism, iron-sulfur clusters and heme biosynthesis, calcium and iron balance, and apoptosis, and are central to the regulation of cellular metabolism and homeostasis [28,29,30,31,32]. These organelles are essential for life, dynamics, and cellular stress responses [29,33]. Mitochondrial dysfunction is closely associated with various phenotypes of aging, such as impaired oxidative phosphorylation (OXPHOS) processes, increased oxidative damage, decreased mitochondrial quality control, decreased metabolic enzyme activity, and changes in mitochondrial morphology, kinetics, and biogenesis [27,34]. Mitochondrial dysfunction can affect host multi-organ systems, resulting in a pathological state known as “mitochondrial disease”. In addition, mitochondrial dysfunction is also associated with many age-related pathological conditions, including neurodegenerative diseases, metabolic diseases, and cardiovascular diseases [35,36,37,38,39,40]. Cellular homeostasis and mitochondrial health require tight regulation between the generation of new cells and the clearance of damaged mitochondria. Recent studies on skin homeostasis and aging have identified the indispensable functions of mitochondria [41] (Figure 2).

A growing body of research has shown that mitochondria have become important players in maintaining skin homeostasis [7]. Mitochondria are at key locations in the skin. While the skin does not have a huge energy requirement compared to other organs such as skeletal muscle, it is vital for effects such as hair growth, immune homeostasis, hormone regulation, pigmentation, and wound healing [42]. UV-caused oxidative stress and its capacity for signaling during extrinsic-induced skin aging have been well documented [43]. However, the importance of mitochondrial damage in UVR-caused skin injury or associated skin manifestations remains to be further clarified. Our review aimed to illustrate the mitochondrial mechanism. Here, we summarize how mitochondria are involved in skin aging and how the opinions can usher in novel insights of mitochondria-targeted approaches that potentially slow down the rate of aging.

### 4.1. Effects of Mitochondria on the Epidermis

It is important to note that the ability required for the sustained regeneration of the skin’s epidermis derives from the adenosine triphosphate (ATP) produced by the mitochondria [44]. Moreover, as keratinocytes gradually differentiate from the basal layer and move up to the basil, mitochondrial structure and function also undergo dynamic changes. In addition, mitochondria-derived ROS can also be regarded as a signal to regulate epidermal differentiation [45]. In terms of metabolism, mitochondria are intermediates that approve the biosynthetic pathways associated with keratin synthesis and lipid metabolism, so mitochondrial homeostasis within keratinocytes is essential for the maintenance of the skin barrier and even host homeostasis [46].

When keratinocytes and slow-proliferating fibroblasts are compared, the accumulation of functional damages ends in keratinocytes for a less pronounced time, and in addition to mitochondrial damage, other mechanisms can affect epidermal integrity [47,48,49]. Alterations in mitochondrial structure are seen during keratinocyte differentiation and persist throughout the skin aging process. The mitochondria number in keratinocytes is significantly higher in the granular layer than in keratinocytes in the spinous layer, and the mitochondria within these different layers form a compact mitochondrial network system [50]. In addition to persistently strong inter-individual variation in mitochondrial proliferation keratinocytes, no age-related differences in mitochondrial numbers were observed to compare cell isolation from young and elderly subjects in vitro, while mitochondrial junction decreased in older keratinocytes with younger ones [50,51].

### 4.2. Effects of Mitochondria on the Dermis

Fibroblasts within the dermis exert an important role in maintaining the skin morphology and function. They are beneficial for the synthesis and keeping of ECM and are positively involved in distinct skin-associated processes, including collagen synthesis and repair. As energy-hungry cells, fibroblasts rely heavily on mitochondria to perform key features, such as ECM synthesis, the synthesis of growth factors, and cell signaling. In addition, mitochondrial function is essential for maintaining fibroblast viability, facilitating proliferation, and enhancing collagen formation, which is vital for keeping skin integrity [52,53,54].

### 4.3. Effects of Mitochondria on the Skin Accessories

The sebaceous gland is an exocrine gland that is beneficial for the release of lipid-abundant sebum and is a key constituent of epidermal defenses, capacity, temperature maintenance, skin strength, and skin microbiota balance [55]. The sebaceous glands are usually linked to hair follicles, a structure consisting of a single pipe leading to the hair follicle, a cluster of secretory cells present at the base of the duct. Glandular cells are mainly made up of a special type of adipocyte that can synthesize and accumulate lipids and participate in other components that constitute sebum [56,57]. Taking into account the active metabolic activity of sebaceous lipids, these cells are rich in highly active mitochondria in their cytoplasm [58].

Mitochondrial dynamics has a vital influence on melanocytes [59]. Studies have shown that genetic and chemical-physical suppression of the mitochondrial fission protein Drp1 results in upregulated melanin generation and mitochondrial lengthening, while reduction of the mitochondrial fusion protein dynein-like GTPase 1 inhibits melanin production, resulting in mitochondrial fragmentation. Correspondingly, mitochondrial fragmentation caused by carbonyl cyanide 3-chlorophenylhydrazone (CCCP) treatment also decreased melanin production. In addition, mitochondrial fission activates the ROS-ERK loop, which leads to the proteasome breakdown of microphthalmia-related transcription elements, further promoting the inhibition of melanin production, favoring the speculation that mitochondrial dynamics exert a vital role in melanin production [60].

Perhaps no other structure is so closely related to the energy of the young and the reduction of the elderly. These associations of the complicated and antagonistic capacities of mitochondria have slowly changed the way we think about the sub-organelles. Mitochondria are seen as more than just energy factories; they are also thought of as a connector for intracellular signal transduction, controllers of innate immunity, and regulators of stem cell activity. These properties, in turn, offer clues to how mitochondria modulate skin aging and skin age-associated disorders [34].

## 5. Structure and Function of Mitochondria

Mitochondria are dynamic organelles within eukaryotic cells and are encased by two layers of membranes. They contain unique DNA that plays a decisive role in maintaining the energy and metabolic requirements of cells. The main task of mitochondria is to produce ATP through cellular metabolism, thus serving as an important source of energy. In addition to energy production, mitochondria exert a key role in regulating cell proliferation, differentiation, calcium, iron balance, and programmed death of cells [61,62]. In addition, mitochondria are associated with keeping electrolyte balance, activating inflammatory responses, and coordinating redox responses [43].

Mitochondria are surrounded by two membranes, inner and outer. This morphological structure constitutes two independent water compartments: the intermembrane space and the mitochondrial matrix, which exert a key role in keeping the cellular function of this cell. The outer membrane exhibits high permeability and consists of various large protein-based pores and positive transporters that are collectively responsible for the transport of proteins, nucleotides, ions, and metabolites between the cytoplasm and the intermembrane space. By contrast, the permeability of the inner membrane is more restricted, and resembles the plasma membrane of a cell. The inner membrane constitutes a large number of folds (cristae) and extends into the matrix of the organelle, in which proteins associated with electron transport and ATP generation. The intermembranous space is located between the two layers of membranes, and its constitution is similar in structure to the cytoplasm. This matrix is encapsulated by an inner membrane and consists of enzymes, mitochondrial DNA (mtDNA), and ribosomes, which are vital for ATP generation. Mitochondrial DNA is encapsulated in a nucleoid, which is translated into vital mitochondrial proteins, which constitute the oxidative phosphorylation complex [61].

The mitochondrial dynamics homeostasis is essential for keeping mitochondrial and cellular balance, and it is regulated by mitochondrial dynamics, mitochondrial biogenesis, and mitophagy. These processes determine the morphology, number, distribution, and location of mitochondria, allowing for optimal cellular energy generation, calcium balance, and other important mitochondrial activity.

Mitochondrial dynamics, mitochondrial biogenesis, and mitophagy are coordinatedly governing and interacting. The mitochondrial fission cleaves the injured mitochondria and cuts them into smaller pieces that can be enclosed by vesicle membranes that provide autophagic content for the mitophagy [63]. The mitochondrial fusion process indirectly governs mitophagy, and when fusion is suppressed, mitochondrial fission further facilitates mitophagy [64]. Meanwhile, mitophagy also influences the fission pathway of mitochondria via binding receptors to related molecules related to mitochondrial dynamics [65,66]. Mitochondrial biogenesis is a closely controlled process that is implicated in a variety of signaling loops and signaling transcription elements. Mitochondrial biogenesis permits cells to satisfy up-regulated energy requirements under different stimuli, eliminating fragmented mitochondria, and is important for cells to adapt to challenges [67]. Once the mitochondria are injured, the mitochondrial network is unable to keep an abundant energy provision [68].

### 5.1. Mitophagy

Mitophagy is a highly selective type of autophagy that permits lysosomal degradation of damaged mitochondria. Mitophagy is associated with fission and fusion, and together they protect mitochondrial homeostasis [69]. Most studies have elucidated the PTEN-caused kinase 1 (PINK1) and Parkin-mediated mitophagy loops [70]. PINK1 localizes to IMM under healthy conditions and is sustainably broken down through proteases. However, when the body is stressed, ΔΨm is depolarized, which in turn leads to the inactivity of proteases and the PINK1 stability. PINK1 is transported by translocase to OMM and accumulates in OMM, where it is autophosphorylated and hires Parkin, subsequently ubiquitinating the substrates, such as Mfn1/2 and voltage-dependent anion channel-1 (VDAC1). The ubiquitinated proteins are then marked and combined with the adaptor protein p62 and eventually undergo light chain 3 (LC3)-mediated clearance of mitochondrial phagocytosis, resulting in degradation of injured mitochondria [71]. Research suggests that Nrf2 also has a vital influence in regulating mitochondrial phagocytosis and maintaining mitochondrial balance. Nrf2 could facilitate mitochondrial phagocytosis and clear damaged mitochondria through the upregulation of p62 and PINK [72]. At the same time, the diminished ability to clear injured mitochondria leads to a large amount of ROS, the unleashing of damage-associated molecular patterns (DAMPs), and the occurrence of inflammation [73,74].

### 5.2. Mitochondrial Dynamics

In order to adapt to alterations in the cellular environment and keep the mitochondria balanced, this process in which mitochondria constantly undergo fission and fusion is known as “mitochondrial dynamics”. Mitochondria maintain the healthy structure and function through a homeostasis of fission and fusion, which is a prerequisite for it to keep physiological capacity. The homeostasis is mediated by various dynamically associated GTPases, which maintain the quantity and quality of mitochondria in parental and daughter cells [75].

Mitochondrial fusion, as a complementary measure, favors the exchange of content between adjacent mitochondria, while fission allows the isolation of damaged mitochondria for the follow-up clear by mitophagy. Mitochondrial proteins (MFN) 1 and 2 are held responsible for the fusion of the outer mitochondrial membrane (OMM); however, the inner mitochondrial membrane (IMM) fusion is dependent on the (optic atrophy 1 (OPA1) protein [76]. In contrast, dyne-associated protein 1 (Drp-1) could be recruited from the cytoplasm to the mitochondrial surface as a central regulator that coordinates fission events [77,78]. The homeostasis of mitochondrial fusion and fission, by regulating mitochondrial morphology, facilitating material exchange, keeping the stability and integrity of mtDNA, and isolating damaged mitochondria, is very important for mitochondrial function [79].

### 5.3. Mitochondrial Biogenesis

Mitochondrial biogenesis is essential for the synthesis of new mitochondria within cells, which is able to be activated by various distinct signals under stress situations and is influenced by toxin increases and mtDNA mutations [80]. Mitochondrial biogenesis is essential for maintaining the quantity and quality of mitochondria involved in the nuclear and mitochondrial genomes. Some transcription elements are associated with the process of mitochondrial biogenesis regulated by physiological stimuli, such as physical activity, nutrient intake, and temperature, etc. Peroxisome proliferation-activating receptor (PPAR)-γ coactivator-1α (1α) regulates the process of co-transcriptional regulators with various transcription elements, including nuclear respiration factors (NRF-1 and NRF-2), mitochondrial transcription factors (Tfam), uncoupling proteins (UCP2), PPARs, thyroid hormones, glucocorticoids, estrogen, and estrogen-associated receptors (ERRs) α and γ [81,82,83]. NRF-1, NRF-2, and Tfam mainly regulate mitochondrial enzyme transcription and mtDNA synthesis [84]. In addition to the transcription elements, two other vital enzymes that are thought to be metabolic receptors that control mitochondrial biogenesis are AMP-activated protein kinase (AMPK) and the mammalian counterpart silencing information regulator 1 (SIRT1). In the case of energy deprivation, AMPK and SIRT1 regulate PGC-1α by phosphorylation and deacetylation, respectively [85]. Some clinical research have indicated that the morphology and mitochondrial numbers in tissues, including the heart, skeletal muscle, and liver tissue, are altered under pathogenic conditions [86].

## 6. Mitochondrial Dysfunction and Aging

Mitochondrial damage and cell aging are two signs of aging and are linked to each other [87]. Mitochondrial dysfunction is characterized as a reduction in the respiration ability of each mitochondrion and a decrease in mitochondrial membrane potential, a process always accompanied by an excess of ROS level, which is thought to be both a consequence and a result of cell aging. This damage has a vital influence on various feedback pathways that cause and maintain the aging manifestations [88]. During cell aging, the increases of damaged mitochondria are caused by elements including excess ROS and mitophagy disorder, which selectively remove dysfunctional mitochondria. As the accumulation of mitochondrial damage gradually increases, mitochondrial dysfunction is further exacerbated, ultimately resulting in downregulated cell bioenergy and increased ROS [89]. This interaction between mitochondrial damage and aging not only leads to cell aging but also has vital influences, affecting cell and organ capacity to play a central role in the broader manifestations of aging and age-associated disorders in organs.

As we age, mitochondria change in morphology, quantity, and OXPHOS activity [90]. Senescence is closely linked to alterations in mitochondrial structure, including hyperfusion or upregulated fragmentation and injury of the mitochondrial network [7,91]. In addition, with age, the oxidative phosphorylation efficiency of mitochondria decreases, resulting in a decrease in ATP production and a decrease in ΔΨM, which impairs cellular energy metabolism. These changes are associated with upregulation of ROS levels during aging, the cumulation of which leads to oxidative stress to mitochondria and cellular ingredients, leading to cell aging [92]. In addition, mitochondrial turnover decreases during aging, which illustrates impaired mitochondrial quality control processes, including downregulation of mitochondrial biogenesis and mitophagy [7]. The mitochondria’s response to stressful events depends on the stimulus and cell type. Notably, the overdose of somatic mtDNA mutations due to internal or external damage impairs mitochondrial capabilities and leads to upregulated ROS formation [93,94].

### 6.1. The Regulatory Roles of Mitochondrial Biogenesis in Aging

As we age, mitochondrial biogenesis gradually downregulates. Mitochondrial biogenesis relies on different signaling cascades to produce normal and intact mitochondria. Generally, PGC-1α is regarded as the intermediate connector of mitochondrial biogenesis. Research has confirmed the importance of PGC-1α in the skin aging process. UVB-irradiated human keratinocyte cells (HaCaT) underwent senescence compared to cells not exposed to UV light, which is characterized by significant downregulation of AMPK phosphorylation levels, SIRT-1, and PGC-1α contents [95]. Guo et al. found that cryptotanshinone (CTS) can promote mitochondrial production in skin cells and ameliorate skin aging via regulating the AMPK-PGC-1α signaling pathway [96]. Similarly, the standardized kaempferia parviflora extract inhibits the intrinsic aging process in human dermal fibroblasts and hairless mice by up-regulating the level of PGC-1α, therefore facilitating mitochondrial biogenesis and ultimately inhibiting cellular senescence and mitochondrial dysfunction [97]. Increased PGC-1α promotes mitochondriogenesis, ameliorates mitochondrial capacities, and delays intrinsic and extrinsic element-caused skin aging.

### 6.2. The Regulatory Roles of Mitophagy in Aging

In senescent cells, mitophagy is activated [12]. Decreased mitophagy and impaired autophagy pathways have been implicated in aging and various age-associated disorders, such as neurodegenerative diseases, cardiac and autoimmune disorders, glucose-metabolic disorders, and tumors [98]. Mitochondrial depolarization is a mark of mitophagy [99]. Activation of DRP1 leads to abnormalities in the morphology and function of mitochondria [100]. Parkin and PINK1 are two protein signs of mitochondrial phagocytosis, and they have a vital influence on mitochondrial capacities [101]. PINK1 continuously accumulates in impaired mitochondria and regulates Parkin, promoting mitophagy [102]. Research showed that when exposed to ultraviolet light, cells initiate mitophagy to clear injured mitochondria, decrease ROS levels, keep photoaging cell balance, suppress mitophagy, disturb photoaging cellular homeostasis, and activate the apoptotic response.

### 6.3. The Regulatory Roles of Mitochondrial Dynamics in Aging

The decrease in mitochondrial biogenesis with age may be the result of an imbalance between mitochondrial fission and fusion pathways, as well as mitochondrial phagocytosis suppression, which favors the elimination of impaired mitochondria [103]. ROS is a family of free radicals that include superoxide anions, hydroxyl groups, peroxyl radicals, and other non-free radicals capable of producing free radicals [104]. While the production of ROS within the cell is an unavoidable process in itself, the cell simultaneously possesses many defense systems to eliminate it to maintain homeostasis. The overproduction of reactive oxygen species is associated with the oxidative damage caused by lipids, DNA, and proteins [105,106]. Previous studies have shown that oxidative stress is linked to a variety of pathophysiological conditions, including aging and age-related diseases [107].

Mitochondria have been the focus of aging research since the introduction of the free radical theory of aging and its later improved version, the mitochondrial aging theories [108,109,110]. Several papers have been introduced in which aged mice genetically deficient in mitochondrial NAD(P)+ transhydrogenase, an enzyme involved in mitochondrial NADPH supply, show impaired motor behavior. According to these papers, it was shown that NNT deficiency interacts with the effects of aging to exacerbate motor dysfunction and cause impaired mitochondrial respiration in oxidative muscles [111,112,113,114]. Among the studies on mitochondrial aging, an increase in ROS can cause cellular deterioration with age, with the theory of mitochondrial aging emphasizing mitochondria as the primary producer of ROS. While the accumulation of oxidative damage occurs with age, evidence on the causal relationship of ROS or mitochondrial ROS as a driver behind aging remains lacking [115].

## 7. Mitochondrial Dysfunction and Skin Aging

**Figure 3:**
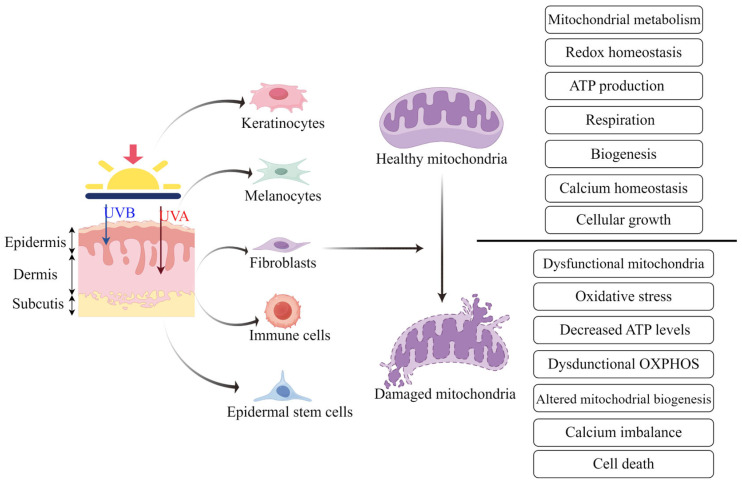
Mitochondrial dysfunction is closely related to skin aging. Ultraviolet exposure (UVB and UVA) induces senescence in different types of cells within the skin, including melanocytes, fibroblasts, immune cells, and epidermal stem cells. Mitochondria in these cells undergo functional and morphological changes, including dysfunctional mitochondria, oxidative stress, decreased ATP levels, dysfunctional OXPHOS, altered mitochondrial biogenesis, calcium imbalance, and cell death.

Skin aging is an intricate process that results in a decrease in the balance and regenerative ability of the organ. Mitochondria are vital organelles that have an important influence on cell mechanisms, including energy synthesis and free radical sustainment. However, mitochondrial dynamics, biogenesis, and degradation processes vary widely in different cells of the skin. It is well known that mitochondrial dysfunction can lead to skin aging and inflammation, further inducing damage to skin physiological functions and the occurrence of related skin diseases. Next, we will summarize the importance of mitochondria in various skin cell species and how damage to mitochondrial structure, functions, and metabolism in the cell contents contributes to the skin aging process (Figure 3).

### 7.1. Mitochondrial Dysfunction and Epidermal Aging

Ultraviolet radiation directly influences the structure and function of the epidermis. Excessive sun exposure can damage keratinocytes, resulting in a decline in the function of skin tissue, which in turn can lead to an imbalance in skin homeostasis [18]. Massive UV exposure can result in necrosis of some epidermal keratinocytes. The damage to cell membrane integrity leads to the emission of cellular contents into the surrounding environment. Adjacent intact keratinocytes absorb the generated contents, such as double-stranded RNA, which promotes toll-like receptors 3 in the endosomes. This process triggers a series of processes of increased inflammatory response and lipid-associated processes [116,117].

UVB exposure of spontaneously transformed aneuploidy immortal keratinocyte cell line cells results in the emission of mitochondria and nuclear-injured DNA, culminating in the promotion of cyclic guanosine monophosphate-adenosine monophosphate synthase/protein stimulator of interferon genes DNA sensor and causing an endogenous immune effect [118]. In addition, long-lasting UV irradiation of spontaneously transformed aneuploidy immortal keratinocytes resulted in a reduction in ΔΨM, a downregulation in mitochondrial mass, and mitochondrial remodeling associated with mitochondria [119,120]. An age-dependent study of mitochondrial networks in youth and older adults showed that keratinocytes in older skin build an obviously more dispersive network with smaller, more compact clusters of mitochondria compared to keratinocytes in younger skin [50].

From the perspective of functional metabolism, aging is linked to alterations in mitochondrial metabolism within capital keratinocytes, including a decrease in OXPHOS [121,122]. The amount of ROS in keratinocytes accumulates with age, while the mitochondrial membrane potential decreases, leading to a metabolic shift from OXPHOS to anaerobic glycolysis in mitochondria. Glycolysis is a compensatory mechanism designed to regulate membrane potential depolarization and gradual decline in cardiolipin levels [121]. The coordination between OXPHOS and glycolysis also controls the homeostasis between stem cell proliferation and differentiation, which in turn determines the fate of epidermal stem cells [123]. An imbalance of low nicotinamide adenine dinucleotide/nicotinamide (NAD+/NAM) ratios also creates keratinocyte differentiation disorders and is linked to the acquisition of premature keratinocyte aging manifestations [122]. They may lead to new therapeutic advances for skin conditions characterized by dysregulated epidermal homeostasis and premature skin aging, such as photoaging.

### 7.2. Mitochondrial Dysfunction and Dermal Aging

During aging, fibroblasts accumulate mitochondrial injury with the loss of mtDNA, which results in structural and functional changes in the ECM and induces an inflammatory response, which in turn promotes the skin wrinkles formation [52,124]. Fibroblasts from aging skin show a number of features linked to aging manifestations and mitochondrial injury. The features include damaged mitochondrial respiration, which reflects upregulated baseline respiration and increased proton leakage, as well as decreased ATP-related respiration and upregulated mitochondrial mass and ROS level [125]. Notably, chronologically aging fibroblasts show a decrease in both the level and activity of mitochondrial complex 2 [126,127]. The latest study by Wedel et al. (2023) reveals a change in mitochondrial fission and fusion pathways in skin fibroblasts during skin aging. The research indicates that a decreased incidence of mitochondrial fission processes or an upregulated perfusion propensity may lead to premature prolongation of mitochondria in aging skin fibroblasts with reduced growth differentiation factor 15 (GDF15) expression [128]. These data were compared to research, in which they elucidated that fibroblasts from centenarian skin maintain mitochondrial bioenergetic capacities via reshaping the mitochondrial network to perfusion. These results highlight the mitochondrial dynamics of senescent skin fibroblasts, suggesting the implications for further investigation to uncover the intricate mechanisms of mitochondrial dynamics during skin aging [129].

### 7.3. Mitochondrial Dysfunction and Adipose Tissue Aging

During the process of skin aging, there are noticeable changes in the quality and distribution of adipose tissue, which is characterized by an upregulation in visceral adipose and a reduction in subcutaneous adipose. Visceral fat tissue is closely linked to impaired metabolic influences, while subcutaneous fat tissue is thought to be metabolically useful. Age-induced peripheral adipose loss may be associated with flaws in lipogenesis and an exacerbation of the inflammatory response to subcutaneous adipose. Mitochondrial damage in adipose tissue during host aging can lead to cell aging, chronic inflammatory response, decreased stem cell activity, and obesity and insulin resistance [130,131]. Aging adipose progenitor cells exhibit injury differentiation ability, which is connected to overdose expression of p53 and p16INK4a, decreased insulin-dependent glucose transport, and dilated adipocyte hypertrophy [132,133].

## 8. Mechanisms of Mitochondrial Dysfunction Leading to Skin Aging

### 8.1. Mitophagy and Skin Aging

Mitophagy is central to mitochondrial quality and quantity control and balance. Once mitophagy is impaired, mitochondrial damage will lead to damaged energy balance and cell injury, which will eventually induce aging and various age-associated disorders, including cancer, cardiovascular disease, metabolic disease, etc. Similarly, mitophagy has been indicated to have a vital influence on the skin aging process. Studies showed that aging keratin-forming cells with high expression of miR-30a showed BNIP3L-dependent mitophagy defects in the final differentiation stage, reflecting that senescent skin development is associated with impaired mitophagy [51]. However, there have been some different voices regarding the negative or positive effects of mitophagy on skin aging. Research showed that mitochondrial dysfunction can be reversed by enhancing mitophagy by restoring imbalance in intracellular homeostasis, further protecting human skin fibroblasts from UVA-caused photoaging [134]. Research also emphasized that the Polygoni Multiflori Radix Preparat (PMRP) enhanced mitophagy by promoting the PINK/parkin signaling loop. However, studies also suggested that mitophagy does not inevitably preserve cells but may induce negative influences and cause apoptotic reactions. In research investigating the protective mechanism of a metabolite derived from intestine microflora, urolithin A in skin aging, centering on the mitophagy process, their data indicated that urolithin A lessened the accumulation of intracellular reactive oxygen species, which promoted the phosphorylation and afterwards nuclear translocation of NRF2, subsequently driving the activation of downstream antioxidative enzymes and restoring mitochondrial function by induction of mitophagy, which was regulated by the SIRT3-FOXO3-PINK1-PARKIN network [135].

### 8.2. Mitochondrial Dynamics and Skin Aging

The process of mitochondrial fusion and fission is involved in the regulation of cellular homeostasis in states of high energy demand, and the interruption of fusion results in the damage of respiratory capacity, which results in mitochondrial damage [136]. The effect of mitochondrial kinetic imbalance on aging is unquestionable and exerts an important role in the skin aging process. Recently, much research has concerned the significance of Drp1 in skin aging, and studies have also shown that inhibiting the expression of Drp1 in cells of skin may slow skin aging. Latest data have found that essential oils from *C. laurie foras* and *C. monspelis* can upregulate the level of the anti-aging gene SIRT1 in normally cultured HaCaT cells. The results are thought to be induced by a decrease of Drp1 level in HaCaT cells [137].

The agonist of the adiponectin receptor, AdipoRon (AR), upregulates phosphorylation of Drp1 Serine 637 via promoting AMP-activated protein kinase (AMPK), preventing mitochondrial translocation, and improving skin inflammatory response-induced skin aging. UV exposure promotes the translocation of Drp1 to mitochondria, causing mitochondrial division and promoting the aging of healthy human keratinocytes (NHK), while suppression of Drp1 delays mitochondrial division. However, Drp1 knockdown did not thoroughly inhibit the likelihood of mitochondrial fission, indicating that Drp1-induced mitochondrial translocation may not be the only pathway by which mitochondrial fission happens in the cells. As of now, there are no clear data on the alterations of mitochondrial fusion protein (Mfn) during skin aging, while Mfn is obviously increased in both skin cancer and melanoma, suggesting that excessive mitochondrial fission caused by Mfn accelerates skin damage [138]. The study revealed that a shift from fusion to a fission network happened in aging skin and that the homeostasis between fission and fusion was damaged [50].

### 8.3. Mitochondrial Biogenesis and Skin Aging

Mitochondrial biogenesis is an important part of mitochondrial quality and quantity monitoring, and AMPK/SIRT1/PGC-1α is a key signaling pathway controlling mitochondrial biogenesis. In a study of myocardial infarction, Guo indicated that the SIRT1/PGC-1α loop plays a vital role in mitochondrial biogenesis [136]. Meanwhile, Sirt1 has been suggested to exhibit anti-aging influences in many cellular models, which are linked to a weakened intracellular response to oxidative stress. Sirt1 modulates the transcriptional viability of PGC-1α by deacetylation. The levels of p-AMPK, Sirt1, and PGC-1α were significantly reduced in HaCaT cells and human foreskin fibroblasts (HFFs) irradiated with ultraviolet light. Excess ultraviolet radiation suppresses the expression of nuclear DNA by damaging it, thereby inhibiting mitochondrial biogenesis. Therefore, these mitochondrial biogenesis-associated proteins have important clinical application value in the diagnosis of skin aging [80].

## 9. Therapeutic Targeting of Skin Mitochondria

In this context, therapeutic drugs that directly aim to mitochondrial function have a wide range of applications in dermatology. They can broadly block harmful products or facilitate cell-specific signaling loops. Topical therapy with a proper vehicle could facilitate permeability of the epidermal layer and dermal-linked tissue, and mitochondria-targeted vehicles or peptides could assist the treatment to achieve its intended location of action to maximize effect.

Given the harmful influences of massively ROS DNA injury and cellular aging [7,139], ROS clearers—naturally occurring or synthetic—have long been regarded as promising treatments to defend mitochondria from injury [140]. For example, CoQ10, a natural ROS clearer that is also regarded as an electron shuttle between Complexes I/II and III of the ETC, is connected to skin aging [141]. New research in mice has indicated that important CoQ10 applications inhibited UV-caused skin aging [142] and upregulated collagen levels and the fibroblast cell quantity [141]. Ubiquitous throughout the body, CoQ10 is an endogenous lipophilic molecule with a central role in cellular energy metabolism—more specifically, the mitochondrial electron transport chain. Additionally, CoQ10 functions as an effective antioxidant molecule involved in the neutralization of reactive species and other free radicals [143]. CoQ10 levels in the skin decrease with age and exposure to UV radiation [141]. Topical supplementation of CoQ10 has been shown to ameliorate signs of skin aging both by stabilizing mitochondrial activity and by exerting antioxidant properties, which typically translates to the regeneration of skin processes involved in cutaneous skin aging [127]. Numerous studies have demonstrated the anti-aging effects of CoQ10 on cultured human dermal fibroblasts [144]. It is a critical ingredient in many anti-aging and regenerative skin creams, and as shown by Knott et al. [145], topical application of two different CoQ10-containing formulas significantly replenished the levels of this antioxidant in the dermal and epidermal layers of older skin (>60 years). Photo-stressed skin benefitted from CoQ10 application on account of a reduction in in situ free radical levels. Schniertshauer et al. [146] further showed that CoQ10 could restore ATP production, prevent mitophagy, and alleviate oxidative stress in aged skin cells. In addition, CoQ10 supplied to the media of the human skin-on-a-chip system resulted in upregulated epidermal and dermal thickness and upregulated keratinocyte marker and collagen I expression [147], a reversal of manifestations of skin aging.

While various natural products have been strengthened for their antioxidant capacity to protect or restore skin aging, as shown in various cosmeceuticals, synthetic substances have also been surveyed for their ROS-clearing viability. For example, MHY553, a PPARα agonist, dose-dependently cleared ROS and suppressed inflammatory factor expression via PPARα expression in the skin [148]. Exhilaratingly, there are over 250 clinical experiments of antioxidants on skin or skin-associated treatments (clinicaltrials.gov, January 2022). As mitochondria are the capital generators of cellular ROS, mitochondria-derived antioxidants, including MitoQ (up to 1000× more beneficial mitochondrial uptake than CoQ10) and tiron (spontaneously localized to the mitochondria without the demand for engineered targeting), have indicated important therapies [149].

## 10. Conclusions and Future Prospects

Skin aging is the noticeable performance of host aging, which is the consequence of the connection of genetic and environmental elements. The effectiveness of skin aging is urgent and vital to keep healthy physiological capacities of the skin and humans’ mental health. The review summarizes the molecular pathways of the vital steps in mitochondrial quality and quantity regulation: mitochondrial dynamics, mitochondrial biogenesis, and mitophagy, and clarifies their influences and underlying mechanisms in skin aging. Recently, the improvement of skin aging induced by environmental elements mainly depended on physical shielding. Antioxidants and enzymes connected to DNA restoration could be supplied to sunblock as ancillary ingredients to partially improve the skin from UV exposure. The supplementation of skin repair or nutritional treatments, including various antioxidants, pharmacological reagents, and active compounds that could obviously ameliorate mitochondrial capacity and improve the metabolic function of the skin, seems to be an effective way. However, basic and clinical studies to prove whether these supplements can be applied in skin anti-aging are still very limited and deserve further investigation.

## Data Availability

Data will be made available on request.

## Abbreviations

DEJ: dermal-epidermal junction; ECM: extracellular matrix; MSCs: mesenchymal stem cells; UV: ultraviolet; UVR: ultraviolet radiation; ROS: reactive oxygen species; OXPHOS: oxidative phosphorylation; CCCP: carbonyl cyanide 3-chlorophenylhydrazone; mtDNA: mitochondrial DNA; PINK1: PTEN-caused kinase 1; VDAC1: voltage-dependent anion channel-1; LC3: light chain 3; DAMPs: damage-associated molecular patterns; MFN: mitochondrial proteins; OMM: outer mitochondrial membrane; IMM: inner mitochondrial membrane; OPA1: optic atrophy 1; Drp-1: dyne-associated protein 1; PPAR-1α: peroxisome proliferation-activating receptor-γ coactivator-1α; NRF: nuclear respiration factors; UCP2: uncoupling proteins; ERRs: estrogen-associated receptors; AMPK: AMP-activated protein kinase; SIRT1: silencing information regulator 1; ΔΨM: mitochondrial membrane potential; HaCaT: human keratinocytes cells; CTS: cryptotanshinone; NAD+/NAM: nicotinamide adenine dinucleotide/nicotinamide; GDF15: growth differentiation factor 15; PMRP: polygoni multiflori radix preparat; AMPK: AMP-activated protein kinase; NHK: normal human keratinocytes; Mfn: mitochondrial fusion protein; HFFs: human foreskin fibroblasts.

## References

[1] Ogrodnik M. (2021). Cellular aging beyond cellular senescence: Markers of senescence prior to cell cycle arrest in vitro and in vivo. Aging Cell.

[2] Summer R., Shaghaghi H., Schriner D., Roque W., Sales D., Cuevas-Mora K., Desai V., Bhushan A., Ramirez M.I., Romero F. (2019). Activation of the mTORC1/PGC-1 axis promotes mitochondrial biogenesis and induces cellular senescence in the lung epithelium. Am. J. Physiol. Cell. Mol. Physiol..

[3] Franco A.C., Aveleira C., Cavadas C. (2022). Skin senescence: Mechanisms and impact on whole-body aging. Trends Mol. Med..

[4] Hu S.C.-S., Lin C.-L., Yu H.-S. (2019). Dermoscopic assessment of xerosis severity, pigmentation pattern and vascular morphology in subjects with physiological aging and photoaging. Eur. J. Dermatol..

[5] D’Uva G., Baci D., Albini A., Noonan D.M. (2018). Cancer chemoprevention revisited: Cytochrome P450 family 1B1 as a target in the tumor and the microenvironment. Cancer Treat. Rev..

[6] Banach K., Kowalska J., Rzepka Z., Beberok A., Rok J., Wrze’sniok D. (2021). The role of UVA radiation in ketoprofen-mediated BRAF-mutant amelanotic melanoma cells death—A study at the cellular and molecular level. Toxicol. In Vitro.

[7] Sreedhar A., Aguilera-Aguirre L., Singh K.K. (2020). Mitochondria in skin health, aging, and disease. Cell Death Dis..

[8] Popov L.D. (2020). Mitochondrial biogenesis: An update. J. Cell. Mol. Med..

[9] Martic I., Papaccio F., Bellei B., Cavinato M. (2023). Mitochondrial dynamics and metabolism across skin cells: Implications for skin homeostasis and aging. Front. Physiol..

[10] Zhang Z., Michniak-Kohn B.B. (2012). Tissue engineered human skin equivalents. Pharmaceutics.

[11] Hill D.S., Robinson N.D., Caley M.P., Chen M., O’Toole E.A., Armstrong J.L., Przyborski S., Lovat P.E. (2015). A novel fully humanized 3D skin equivalent to model early melanoma invasion. Mol. Cancer Ther..

[12] Wang H., Guo B., Hui Q., Lin F., Tao K. (2020). CO_2_ lattice laser reverses skin aging caused by UVB. Aging.

[13] Kim J.H., Jeong H.D., Song M.J., Lee D.H., Chung J.H., Lee S.T. (2022). SOD3 suppresses the expression of MMP-1 and increases the integrity of extracellular matrix in fibroblasts. Antioxidants.

[14] Hofmann E., Schwarz A., Fink J., Kamolz L.P., Kotzbeck P. (2023). Modelling the complexity of human skin in vitro. Biomedicines.

[15] Abdallah F., Mijouin L., Pichon C. (2017). Skin immune landscape: Inside and outside the organism. Mediat. Inflamm..

[16] Trevor L.V., Riches-Suman K., Mahajan A.L., Thornton M.J. (2020). Adipose tissue: A source of stem cells with potential for regenerative therapies for wound healing. J. Clin. Med..

[17] Mancuso P., Bouchard B. (2019). The impact of aging on adipose function and adipokine synthesis. Front. Endocrinol..

[18] Slominski A., Wortsman J. (2000). Neuroendocrinology of the skin. Endocr. Rev..

[19] Naidoo K., Hanna R., Birch-Machin M.A. (2018). What is the role of mitochondrial dysfunction in skin photoaging?. Exp. Dermatol..

[20] Farage M.A., Miller K.W., Elsner P., Maibach H.I. (2008). Intrinsic and extrinsic factors in skin ageing: A review. Int. J. Cosmet. Sci..

[21] Yaar M., Eller M.S., Gilchrest B.A. (2002). Fifty years of skin aging. J. Investig. Dermatol. Symp. Proc..

[22] Kim D.J., Iwasaki A., Chien A.L., Kang S. (2022). UVB-mediated DNA damage induces matrix metalloproteinases to promote photoaging in an AhR- and SP1-dependent manner. JCI Insight.

[23] Kim H., Jang J., Song M.J., Park C.H., Lee D.H., Lee S.H., Chung J.H. (2022). Inhibition of matrix metalloproteinase expression by selective clearing of senescent dermal fibroblasts attenuates ultraviolet-induced photoaging. Biomed. Pharmacother..

[24] Moriyama M., Moriyama H., Uda J., Kubo H., Nakajima Y., Goto A., Morita T., Hayakawa T. (2017). BNIP3 upregulation via stimulation of ERK and JNK activity is required for the protection of keratinocytes from UVB-induced apoptosis. Cell Death Dis..

[25] Brand R.M., Wipf P., Durham A., Epperly M.W., Greenberger J.S., Falo L.D. (2018). Targeting mitochondrial oxidative stress to mitigate UV-induced skin damage. Front. Pharmacol..

[26] Valejo Coelho M.M., Matos T.R., Apetato M. (2016). The dark side of the light: Mechanisms of photocarcinogenesis. Clin. Dermatol..

[27] Kauppila T.E.S., Kauppila J.H.K., Larsson N.G. (2017). Mammalian mitochondria and aging: An update. Cell Metab..

[28] Nicholls D.G. (2005). Mitochondria and calcium signaling. Cell Calcium..

[29] Liu Z., Butow R.A. (2006). Mitochondrial retrograde signaling. Annu. Rev. Genet..

[30] Nilsson R., Schultz I.J., Pierce E.L., Soltis K.A., Naranuntarat A., Ward D.M., Baughman J.M., Paradkar P.N., Kingsley P.D., Culotta V.C. (2009). Discovery of genes essential for heme biosynthesis through large-scale gene expression analysis. Cell Metab..

[31] Wallace D.C., Fan W., Procaccio V. (2010). Mitochondrial energetics and therapeutics. Annu. Rev. Pathol..

[32] Srivastava S. (2016). Emerging therapeutic roles for NAD^+^ metabolism in mitochondrial and age-related disorders. Clin. Transl. Med..

[33] Butow R.A., Avadhani N.G. (2004). Mitochondrial signaling: The retrograde response. Mol. Cell.

[34] Sun N., Youle R.J., Finkel T. (2016). The mitochondrial basis of aging. Mol. Cell.

[35] Petersen K.F., Befroy D., Dufour S., Dziura J., Ariyan C., Rothman D.L., DiPietro L., Cline G.W., Shulman G.I. (2003). Mitochondrial dysfunction in the elderly: Possible role in insulin resistance. Science.

[36] Lowell B.B., Shulman G.I. (2005). Mitochondrial dysfunction and type 2 diabetes. Science.

[37] Wallace D.C. (2012). Mitochondria and cancer. Nat. Rev. Cancer.

[38] Lane R.K., Hilsabeck T., Rea S.L. (2015). The role of mitochondrial dysfunction in age-related diseases. Biochim. Biophys. Acta.

[39] Tocchi A., Quarles E.K., Basisty N., Gitari L., Rabinovitch P.S. (2015). Mitochondrial dysfunction in cardiac aging. Biochim. Biophys. Acta.

[40] Li Q.O.Y., Soro-Arnaiz I., Aragonés J. (2017). Age-dependent obesity and mitochondrial dysfunction. Adipocyte.

[41] Ahn C.S.M., Etallo C.M. (2015). Mitochondria as biosynthetic factories for cancer proliferation. Cancer Metab..

[42] Stout R., Birch-Machin M. (2019). Mitochondria’s role in skin ageing. Biology.

[43] Lin S.J., Kaeberlein M., Andalis A.A., Sturtz L.A., Defossez P.A., Culotta V.C., Fink G.R., Guarente L. (2002). Calorie restriction extends Saccharomyces cerevisiae lifespan by increasing respiration. Nature.

[44] Kirkinezos I.G., Moraes C.T. (2001). Reactive oxygen species and mitochondrial diseases. Semin. Cell Dev. Biol..

[45] Hamanaka R.B., Chandel N.S. (2013). Mitochondrial metabolism as a regulator of keratinocyte differentiation. Cell Logist..

[46] Trompette A., Pernot J., Perdijk O., Alqahtani R.A.A., Domingo J.S., Camacho-Muñoz D., Wong N.C., Kendall A.C., Wiederkehr A., Nicod L.P. (2022). Gut-derived short-chain fatty acids modulate skin barrier integrity by promoting keratinocyte metabolism and differentiation. Mucosal. Immunol..

[47] Yaar M., Gilchrest B.A. (2001). Ageing and photoageing of keratinocytes and melanocytes. Clin. Exp. Dermatol..

[48] Farage M.A., Miller K.W., Elsner P., Maibach H.I. (2013). Characteristics of the aging skin. Adv. Wound Care.

[49] Khalid K.A., Nawi A.F.M., Zulkifli N., Barkat M.A., Hadi H. (2022). Aging and wound healing of the skin: A review of clinical and pathophysiological hallmarks. Life.

[50] Mellem D., Sattler M., Pagel-Wolff S., Jaspers S., Wenck H., Rübhausen M.A., Fischer F. (2017). Fragmentation of the mitochondrial network in skin in vivo. PLoS ONE.

[51] Chevalier F.P., Rorteau J., Ferraro S., Martin L.S., Gonzalez-Torres A., Berthier A., El Kholti N., Lamartine J. (2022). MiR-30a-5p alters epidermal terminal differentiation during aging by regulating BNIP3L/NIX-dependent mitophagy. Cells.

[52] Krutmann J., Schroeder P. (2009). Role of mitochondria in photoaging of human skin: The defective powerhouse model. J. Investig. Dermatol. Symp. Proc..

[53] Katsuyama Y., Yamawaki Y., Sato Y., Muraoka S., Yoshida M., Okano Y., Masaki H. (2022). Decreased mitochondrial function in UVA-irradiated dermal fibroblasts causes the insufficient formation of type I collagen and fibrillin-1 fibers. J. Dermatol. Sci..

[54] Yanes B., Rainero E. (2022). The interplay between cell-extracellular matrix interaction and mitochondria dynamics in cancer. Cancers.

[55] Ahmed N.S., Foote J.B., Singh K.K. (2022). Impaired mitochondria promote aging-associated sebaceous gland dysfunction and pathology. Am. J. Pathol..

[56] Lovászi M., Szegedi A., Zouboulis C.C., Törőcsik D. (2017). Sebaceous-immunobiology is orchestrated by sebum lipids. Derm.-Endocrinol..

[57] Wikramanayake T.C., Nicu C., Gherardini J., Mello A.C.G.C.V., Chéret J., Paus R. (2022). Mitochondrially localized MPZL3 functions as a negative regulator of sebaceous gland size and sebocyte proliferation. J. Investig. Dermatol..

[58] Schneider M.R., Paus R. (2010). Sebocytes, multifaceted epithelial cells: Lipid production and holocrine secretion. Int. J. Biochem. Cell Biol..

[59] Yu R., Lendahl U., Nistér M., Zhao J. (2020). Regulation of mammalian mitochondrial dynamics: Opportunities and challenges. Front. Endocrinol..

[60] Kim E.S., Park S.J., Goh M.J., Na Y.J., Jo D.S., Jo Y.K., Shin J.H., Choi E.S., Lee H.K., Kim J.Y. (2014). Mitochondrial dynamics regulate melanogenesis through proteasomal degradation of MITF via ROS-ERK activation. Pigment Cell Melanoma Res..

[61] Vasileiou P.V.S., Evangelou K., Vlasis K., Fildisis G., Panayiotidis M.I., Chronopoulos E., Passias P.G., Kouloukoussa M., Gorgoulis V.G., Havaki S. (2019). Mitochondrial homeostasis and cellular senescence. Cells.

[62] Lee S.E., Kang Y.C., Kim Y., Kim S., Yu S.H., Park J.H., Kim I.H., Kim H.Y., Han K., Lee H.K. (2022). Preferred migration of mitochondria toward cells and tissues with mitochondrial damage. Int. J. Mol. Sci..

[63] Li W., Li H., Zheng L., Xia J., Yang X., Men S., Yuan Y., Fan Y. (2023). Ginsenoside CK improves skeletal muscle insulin resistance by activating DRP1/PINK1-mediated mitophagy. Food Funct..

[64] Ma F., Li H., Huo H., Han Q., Liao J., Zhang H., Li Y., Pan J., Hu L., Guo J. (2023). N-acetyl-L-cysteine alleviates FUNDC1-mediated mitophagy by regulating mitochondrial dynamics in type 1 diabetic nephropathy canine. Life Sci..

[65] Al-Bari M.A.A., Xu P. (2020). Molecular regulation of autophagy machinery by mTOR-dependent and -independent pathways. Ann. N. Y. Acad. Sci..

[66] Shirihai O.S., Song M., Dorn G.W. (2015). How mitochondrial dynamism orchestrates mitophagy. Circ. Res..

[67] Shi R., Guberman M., Kirshenbaum L.A. (2018). Mitochondrial quality control: The role of mitophagy in aging. Trends Cardiovasc. Med..

[68] Zhu N., Xu M.H., Li Y. (2022). Bioactive oligopeptides from ginseng (*Panax ginseng* Meyer) suppress oxidative stress-induced senescence in fibroblasts via NAD+/SIRT1/PGC-1α signaling pathway. Nutrients.

[69] Pickles S., Vigié P., Youle R.J. (2018). Mitophagy and quality control mechanisms in mitochondrial maintenance. Curr. Biol..

[70] Matsuda N., Sato S., Shiba K., Okatsu K., Saisho K., Gautier C.A., Sou Y.S., Saiki S., Kawajiri S., Sato F. (2010). PINK1 stabilized by mitochondrial depolarization recruits parkin to damaged mitochondria and activates latent parkin for mitophagy. J. Cell Biol..

[71] Geisler S., Holmström K.M., Skujat D., Fiesel F.C., Rothfuss O.C., Kahle P.J., Springer W. (2010). PINK1/parkin-mediated mitophagy is dependent on VDAC1 and p62/SQSTM1. Nat. Cell Biol..

[72] Ryoo I.G., Kwak M.K. (2018). Regulatory crosstalk between the oxidative stress-related transcription factor Nfe2l2/Nrf2 and mitochondria. Toxicol. Appl. Pharmacol..

[73] Niu Q., Chen J., Xia T., Li P., Zhou G., Xu C., Zhao Q., Dong L., Zhang S., Wang A. (2018). Excessive ER stress and the resulting autophagic flux dysfunction contribute to fluoride-induced neurotoxicity. Environ. Pollut..

[74] Meyer J.N., Leuthner T.C., Luz A.L. (2017). Mitochondrial fusion, fission, and mitochondrial toxicity. Toxicology.

[75] Morton H., Kshirsagar S., Orlov E., Bunquin L.E., Sawant N., Boleng L., George M., Basu T., Ramasubramanian B., Pradeepkiran J.A. (2021). Defective mitophagy and synaptic degeneration in Alzheimer’s disease: Focus on aging, mitochondria and synapse. Free Radic. Biol. Med..

[76] Jugé R., Breugnot J., Da Silva C., Bordes S., Closs B., Aouacheria A. (2016). Quantification and characterization of UVB-induced mitochondrial fragmentation in normal primary human keratinocytes. Sci. Rep..

[77] Pagliuso A., Cossart P., Stavru F. (2018). The ever-growing complexity of the mitochondrial fission machinery. Cell. Mol. Life Sci..

[78] Gupta D., Abdullah T.S. (2022). Regulation of mitochondrial dynamics in skin: Role in pathophysiology. Int. J. Dermatol..

[79] Chan D.C. (2020). Mitochondrial dynamics and its involvement in disease. Annu. Rev. Pathol. Mech. Dis..

[80] Zhang C., Gao X., Li M., Yu X., Huang F., Wang Y., Yan Y., Zhang H., Shi Y., He X. (2023). The role of mitochondrial quality surveillance in skin aging: Focus on mitochondrial dynamics, biogenesis and mitophagy. Ageing Res. Rev..

[81] Ventura-Clapier R., Garnier A., Veksler V. (2008). Transcriptional control of mitochondrial biogenesis: The central role of PGC-1alpha. Cardiovasc. Res..

[82] Jornayvaz F.R., Shulman G.I. (2010). Regulation of mitochondrial biogenesis. Essays Biochem..

[83] Hock M.B., Kralli A. (2009). Transcriptional control of mitochondrial biogenesis and function. Annu. Rev. Physiol..

[84] Virbasius J.V., Scarpulla R.C. (1994). Activation of the human mitochondrial transcription factor A gene by nuclear respiratory factors: A potential regulatory link between nuclear and mitochondrial gene expression in organelle biogenesis. Proc. Natl. Acad. Sci. USA.

[85] Reznick R.M., Shulman G.I. (2006). The role of AMP-activated protein kinase in mitochondrial biogenesis. J. Physiol..

[86] Ren J., Pulakat L., Whaley-Connell A., Sowers J.R. (2010). Mitochondrial biogenesis in the metabolic syndrome and cardiovascular disease. J. Mol. Med..

[87] Miwa S., Kashyap S., Chini E., von Zglinicki T. (2022). Mitochondrial dysfunction in cell senescence and aging. J. Clin. Investig..

[88] Chapman J., Fielder E., Passos J.F. (2019). Mitochondrial dysfunction and cell senescence: Deciphering a complex relationship. FEBS Lett..

[89] Ghosh-Choudhary S.K., Liu J., Finkel T. (2021). The role of mitochondria in cellular senescence. FASEB J..

[90] Shigenaga M.K., Hagen T.M., Ames B.N. (1994). Oxidative damage and mitochondrial decay in aging. Proc. Natl. Acad. Sci. USA.

[91] Liesa M., Shirihai O.S. (2013). Mitochondrial dynamics in the regulation of nutrient utilization and energy expenditure. Cell Metab..

[92] Gao X., Yu X., Zhang C., Wang Y., Sun Y., Sun H., Zhang H., Shi Y., He X. (2022). Telomeres and mitochondrial metabolism: Implications for cellular senescence and age-related diseases. Stem Cell Rev. Rep..

[93] Zorov D.B., Vorobjev I.A., Popkov V.A., Babenko V.A., Zorova L.D., Pevzner I.B., Silachev D.N., Zorov S.D., Andrianova N.V., Plotnikov E.Y. (2019). Lessons from the discovery of mitochondrial fragmentation (fission): A review and update. Cells.

[94] Cavinato M., Madreiter-Sokolowski C.T., Büttner S., Schosserer M., Zwerschke W., Wedel S., Grillari J., Graier W.F., Jansen-Dürr P. (2021). Targeting cellular senescence based on interorganelle communication, multilevel proteostasis, and metabolic control. FEBS J..

[95] Jing R., Guo K., Zhong Y., Wang L., Zhao J., Gao B., Ye Z., Chen Y., Li X., Xu N. (2021). Protective effects of fucoidan purified from Undaria pinnatifida against UV-irradiated skin photoaging. Ann. Transl. Med..

[96] Guo K., Liu R., Jing R., Wang L., Li X., Zhang K., Fu M., Ye J., Hu Z., Zhao W. (2022). Cryptotanshinone protects skin cells from ultraviolet radiation-induced photoaging via its antioxidant effect and by reducing mitochondrial dysfunction and inhibiting apoptosis. Front. Pharmacol..

[97] Park J.E., Woo S.W., Kim M.B., Kim C., Hwang J.K. (2017). Standardized kaempferia parviflora extract inhibits intrinsic aging process in human dermal fibroblasts and hairless mice by inhibiting cellular senescence and mitochondrial dysfunction. Evid. Based Complement. Alternat. Med..

[98] Palikaras K., Daskalaki I., Markaki M., Tavernarakis N. (2017). Mitophagy and age-related pathologies: Development of new therapeutics by targeting mitochondrial turnover. Pharmacol. Ther..

[99] Mengus C., Neutzner M., Bento A.C.P.F., Bippes C.C., Kohler C., Decembrini S., Häusel J., Hemion C., Sironi L., Frank S. (2022). VCP/p97 cofactor UBXN1/SAKS1 regulates mitophagy by modulating MFN2 removal from mitochondria. Autophagy.

[100] Ko H.J., Tsai C.Y., Chiou S.J., Lai Y.L., Wang C.H., Cheng J.T., Chuang T.H., Huang C.F., Kwan A.L., Loh J.K. (2021). The phosphorylation status of Drp1-Ser637 by PKA in mitochondrial fission modulates mitophagy via PINK1/Parkin to exert multipolar spindles assembly during mitosis. Biomolecules.

[101] Barazzuol L., Giamogante F., Brini M., Calì T. (2020). PINK1/Parkin mediated mitophagy, Ca^2+^ signalling, and ER-mitochondria contacts in Parkinson’s disease. Int. J. Mol. Sci..

[102] Sarraf S.A., Sideris D.P., Giagtzoglou N., Ni L., Kankel M.W., Sen A., Bochicchio L.E., Huang C.H., Nussenzweig S.C., Worley S.H. (2019). PINK1/Parkin influences cell cycle by sequestering TBK1 at damaged mitochondria, inhibiting mitosis. Cell Rep..

[103] Chistiakov D.A., Sobenin I.A., Revin V.V., Orekhov A.N., Bobryshev Y.V. (2014). Mitochondrial aging and age-related dysfunction of mitochondria. BioMed Res. Int..

[104] Halliwell B. (2006). Reactive species and antioxidants. Redox biology is a fundamental theme of aerobic life. Plant Physiol..

[105] Halliwell B., Gutteridge J.M.C. (2007). Free Radicals in Biology and Medicine.

[106] Finkel T., Holbrook N.J. (2000). Oxidants, oxidative stress and the biology of ageing. Nature.

[107] Thanan R., Oikawa S., Hiraku Y., Ohnishi S., Ma N., Pinlaor S., Yongvanit P., Kawanishi S., Murata M. (2015). Oxidative stress and its significant roles in neurodegenerative diseases and cancer. Int. J. Mol. Sci..

[108] Harman D. (1956). Aging: A theory based on free radical and radiation chemistry. J. Gerontol..

[109] Harman D. (1972). The biologic clock: The mitochondria?. J. Am. Geriatr. Soc..

[110] Alexeyev M.F. (2009). Is there more to aging than mitochondrial DNA and reactive oxygen species?. FEBS J..

[111] Francisco A., Engel D.F., Figueira T.R., Rogério F., de Bem A.F., Castilho R.F. (2020). Mitochondrial NAD(P)^+^ transhydrogenase is unevenly distributed in different brain regions, and its loss causes depressive-like behavior and motor dysfunction in mice. Neuroscience.

[112] Navarro C.D.C., Francisco A., Costa E.F.D., Dalla Costa A.P., Sartori M.R., Bizerra P.F.V., Salgado A.R., Figueira T.R., Vercesi A.E., Castilho R.F. (2024). Aging-dependent mitochondrial bioenergetic impairment in the skeletal muscle of NNT-deficient mice. Exp. Gerontol..

[113] Kadoguchi T., Takada S., Yokota T., Furihata T., Matsumoto J., Tsuda M., Mizushima W., Fukushima A., Okita K., Kinugawa S. (2018). Deletion of NAD(P)H oxidase 2 prevents angiotensin II-induced skeletal muscle atrophy. BioMed Res. Int..

[114] Figueira T.R., Francisco A., Ronchi J.A., Dos Santos G.R.R.M., Santos W.D., Treberg J.R., Castilho R.F. (2021). NADPH supply and the contribution of NAD(P)^+^ transhydrogenase (NNT) to H_2_O_2_ balance in skeletal muscle mitochondria. Arch. Biochem. Biophys..

[115] Bokov A., Chaudhuri A., Richardson A. (2004). The role of oxidative damage and stress in aging. Mech. Ageing Dev..

[116] Bernard J.J., Cowing-Zitron C., Nakatsuji T., Muehleisen B., Muto J., Borkowski A.W., Martinez L., Greidinger E.L., Yu B.D., Gallo R.L. (2012). Ultraviolet radiation damages self noncoding RNA and is detected by TLR3. Nat. Med..

[117] Borkowski A.W., Gallo R.L. (2014). UVB radiation illuminates the role of TLR3 in the epidermis. J. Investig. Dermatol..

[118] Li C., Liu W., Wang F., Hayashi T., Mizuno K., Hattori S., Fujisaki H., Ikejima T. (2021). DNA damage-triggered activation of cGAS-STING pathway induces apoptosis in human keratinocyte HaCaT cells. Mol. Immunol..

[119] Kelly J., Murphy J.E. (2016). Mitochondrial tolerance to single and repeat exposure to simulated sunlight in human epidermal and dermal skin cells. J. Photochem. Photobiol. B.

[120] Bellei B., Picardo M. (2020). Premature cell senescence in human skin: Dual face in chronic acquired pigmentary disorders. Ageing Res. Rev..

[121] Dumas M., Maftah A., Bonte F., Ratinaud M.H., Meybeck A., Julien R. (1995). Flow cytometric analysis of human epidermal cell ageing using two fluorescent mitochondrial probes. Comptes Rendus L’academie Sci. Ser. III.

[122] Tan C.L., Chin T., Tan C.Y.R., Rovito H.A., Quek L.S., Oblong J.E., Bellanger S. (2019). Nicotinamide metabolism modulates the proliferation/differentiation balance and senescence of human primary keratinocytes. J. Investig. Dermatol..

[123] Folmes C.D., Dzeja P.P., Nelson T.J., Terzic A. (2012). Metabolic plasticity in stem cell homeostasis and differentiation. Cell Stem Cell.

[124] Ho C.Y., Dreesen O. (2021). Faces of cellular senescence in skin aging. Mech. Ageing Dev..

[125] Rorteau J., Chevalier F.P., Bonnet S., Barthélemy T., Lopez-Gaydon A., Martin L.S., Bechetoille N., Lamartine J. (2022). Maintenance of chronological aging features in culture of normal human dermal fibroblasts from old donors. Cells.

[126] Bowman A., Birch-Machin M.A. (2016). Age-dependent decrease of mitochondrial complex II activity in human skin fibroblasts. J. Investig. Dermatol..

[127] Schniertshauer D., Gebhard D., Bergemann J. (2018). Age-dependent loss of mitochondrial function in epithelial tissue can be reversed by coenzyme Q10. J. Aging Res..

[128] Wedel S., Martic I., Guerrero Navarro L., Ploner C., Pierer G., Jansen-Dürr P., Cavinato M. (2023). Depletion of growth differentiation factor 15 (GDF15) leads to mitochondrial dysfunction and premature senescence in human dermal fibroblasts. Aging Cell.

[129] Sgarbi G., Matarrese P., Pinti M., Lanzarini C., Ascione B., Gibellini L., Dika E., Patrizi A., Tommasino C., Capri M. (2014). Mitochondria hyperfusion and elevated autophagic activity are key mechanisms for cellular bioenergetic preservation in centenarians. Aging.

[130] Woo C.Y., Jang J.E., Lee S.E., Koh E.H., Lee K.U. (2019). Mitochondrial dysfunction in adipocytes as a primary cause of adipose tissue inflammation. Diabetes Metab. J..

[131] Ou M.Y., Zhang H., Tan P.C., Zhou S.B., Li Q.F. (2022). Adipose tissue aging: Mechanisms and therapeutic implications. Cell Death Dis..

[132] Krstic J., Reinisch I., Schupp M., Schulz T.J., Prokesch A. (2018). p53 functions in adipose tissue metabolism and homeostasis. Int. J. Mol. Sci..

[133] de Lange P., Lombardi A., Silvestri E., Cioffi F., Giacco A., Iervolino S., Petito G., Senese R., Lanni A., Moreno M. (2023). Physiological approaches targeting cellular and mitochondrial pathways underlying adipose organ senescence. Int. J. Mol. Sci..

[134] Liu W., Yan F., Xu Z., Chen Q., Ren J., Wang Q., Chen L., Ying J., Liu Z., Zhao J. (2022). Urolithin A protects human dermal fibroblasts from UVA-induced photoaging through NRF2 activation and mitophagy. J. Photochem. Photobiol. B..

[135] Chen L., Qin Y., Liu B., Gao M., Li A., Li X., Gong G. (2022). PGC-1α-mediated mitochondrial quality control: Molecular mechanisms and implications for heart failure. Front. Cell Dev. Biol..

[136] Guo Z., Fan D., Liu F.Y., Ma S.Q., An P., Yang D., Wang M.Y., Yang Z., Tang Q.Z. (2022). NEU1 Regulates Mitochondrial Energy Metabolism and Oxidative Stress Post-myocardial Infarction in Mice via the SIRT1/PGC-1 Alpha Axis. Front. Cardiovasc. Med..

[137] Ledrhem M., Nakamura M., Obitsu M., Hirae K., Kameyama J., Bouamama H., Gadhi C., Katakura Y. (2022). Essential oils derived from cistus species activate mitochondria by inducing SIRT1 expression in human keratinocytes, leading to senescence inhibition. Molecules.

[138] Soares C.D., de Lima Morais T.M., Mariano F.V., Altemani A., Corrêa M.B., Reis R.R.D.D., Amorim L.S., Ferreira S.M.S., de Almeida O.P., Carlos R. (2019). Expression of mitochondrial dynamics markers during melanoma progression: Comparative study of head and neck cutaneous and mucosal melanomas. J. Oral Pathol. Med..

[139] Chen G., Kroemer G., Kepp O. (2020). Mitophagy: An emerging role in aging and age-associated diseases. Front. Cell Dev. Biol..

[140] Russell O.M., Gorman G.S., Lightowlers N., Turnbull D.M. (2020). Mitochondrial diseases: Hope for the future. Cell.

[141] Ayunin Q., Miatmoko A., Soeratri W., Erawati T., Susanto J., Legowo D. (2022). Improving the anti-ageing activity of coenzyme Q10 through protransfersome-loaded emulgel. Sci. Rep..

[142] Wu H., Zhong Z., Lin S., Qiu C., Xie P., Lv S., Cui L., Wu T. (2020). Coenzyme Q_10_ sunscreen prevents progression of ultraviolet-induced skin damage in mice. BioMed Res. Int..

[143] Sguizzato M., Mariani P., Spinozzi F., Benedusi M., Cervellati F., Cortesi R., Drechsler M., Prieux R., Valacchi G., Esposito E. (2020). Ethosomes for Coenzyme Q10 cutaneous administration: From design to 3D skin tissue evaluation. Antioxidants.

[144] Hoppe U., Bergemann J., Diembeck W., Ennen J., Gohla S., Harris I., Jacob J., Kielholz J., Mei W., Pollet D. (1999). Coenzyme Q10, a cutaneous antioxidant and energizer. Biofactors.

[145] Knott A., Achterberg V., Smuda C., Mielke H., Sperling G., Dunckelmann K., Vogelsang A., Krüger A., Schwengler H., Behtash M. (2015). Topical treatment with coenzyme Q10-containing formulas improves skin’s Q10 level and provides antioxidative effects. Biofactors.

[146] Schniertshauer D., Müller S., Mayr T., Sonntag T., Gebhard D., Bergemann J. (2016). Accelerated Regeneration of ATP Level after Irradiation in Human Skin Fibroblasts by Coenzyme Q10. Photochem. Photobiol..

[147] Kim J., Kim K., Sung G.Y. (2020). Coenzyme Q10 efficacy test for human skin equivalents using a pumpless skin-on-a-chip system. Int. J. Mol. Sci..

[148] Jung H.J., Kim S.M., Kim D.H., Bang E., Kang D., Lee S., Chun P., Moon H.R., Chung H.Y. (2021). 2,4-Dihydroxyphenyl-benzo[d] thiazole (MHY553), a synthetic PPARα agonist, decreases age-associated inflammatory responses through PPARα activation and RS scavenging in the skin. Exp. Gerontol..

[149] Oyewole A.O., Birch-Machin M.A. (2015). Mitochondria-targeted antioxidants. FASEB J..

